# Selective Electron Beam Melting (SEBM) of Pure Tungsten: Metallurgical Defects, Microstructure, Texture and Mechanical Properties

**DOI:** 10.3390/ma15031172

**Published:** 2022-02-03

**Authors:** Xin Ren, Hui Peng, Jingli Li, Hailin Liu, Liming Huang, Xin Yi

**Affiliations:** 1 Department of Mechanics and Engineering Science, College of Engineering, Peking University, Beijing 100871, China; xinren_1986@163.com (X.R.); lijingli@pku.edu.cn (J.L.); 1601111544@pku.edu.cn (H.L.); liming_huang@pku.edu.cn (L.H.); 2 Research Institute for Frontier Science, Beihang University, Beijing 100191, China; penghui@buaa.edu.cn

**Keywords:** pure tungsten, selective electron beam melting (SEBM), porosity, microstructure, mechanical properties

## Abstract

Effects of processing parameters on the metallurgical defects, microstructure, texture, and mechanical properties of pure tungsten samples fabricated by selective electron beam melting are investigated. SEBM-fabricated bulk tungsten samples with features of lack of fusion, sufficient fusion, and over-melting are examined. For samples upon sufficient fusion, an ultimate compressive strength of 1.76 GPa is achieved at the volumetric energy density of 900 J/mm^3^–1000 J/mm^3^. The excellent compressive strength is higher and the associated volumetric energy density is significantly lower than corresponding reported values in the literature. The average relative density of SEBM-fabricated samples is 98.93%. No microcracks, but only pores with diameters of few tens of micrometers, are found in SEBM-ed tungsten samples of sufficient fusion. Properties of samples by SEBM and selective laser melting (SLM) have also been compared. It is found that SLM-fabricated samples exhibit inevitable microcracks, and have a significantly lower ultimate compressive strength and a slightly lower relative density of 98.51% in comparison with SEBM-ed samples.

## 1. Introduction

Tungsten has a high melting point, a high density, a low erosion tendency, high thermal stress resistance, and high thermal conductivity, and exhibits low swelling and low tritium retention. Owing to these superior thermophysical properties, tungsten and tungsten alloys are considered the most promising candidate materials for plasma facing components in nuclear reactors [1], and have also been attractive for industrial, aerospace, and medical applications such as high temperature furnaces and X-ray shielding [2]. On the other hand, tungsten materials show processing difficulties and have limited engineering applications due to the inherent features of high-melting point, brittleness at room temperature, and high temperature oxidation behaviors [3,4].

Selective laser melting (SLM) and selective electron beam manufacturing (SEBM) are two important representatives of powder bed based additive manufacturing processes for forming near-net shaped components of metals. They not only offer new feasible processing methods for refractory metals, but also liberate the design freedom and significantly expand the engineering application scope of refractory metals.

Rapid progress has been made in understanding the processing-microstructure-properties relationships of pure tungsten samples fabricated by SLM [3,4,5,6,7]. For example, the SLM-fabricated bulk pure tungsten samples of high relative densities of 95–98.51% can achieve an ultimate compressive strength up to 1.01 GPa after heat treatment [7,8]. Experimental observations also indicate that microcracks owing to high residual stresses induced by high temperature gradients in SLM-ed samples are inevitable [4,5,6,7,8,9,10,11]. In SLM-ed samples, one can also find pores whose formation is most likely attributed to the entrapped protective inert gas (e.g., argon) by the Marangoni effect [7,12].

In contrast to SLM with laser power on the order of hundreds of Watts, the electron beam power of SEBM can reach as high as 3 kW, much higher energy input than SLM [13]. In SEBM, preheating the substrate and every powder layer by the defocused electron beam can significantly minimize the residual stress in the fabricated components. The SEBM process works under a vacuum condition which forms a nearly perfect protection against oxidization and gas contamination [14,15]. Owing to these features, SEBM is more suitable than SLM for fabricating refractory metal components and brittle materials with affinity to gases such as oxygen at high temperature [16,17,18,19].

In comparison with studies of SLM-ed pure tungsten samples [3,4,5,6,7], few studies on the fabrication of pure tungsten by SEBM have been reported in the literature [20]. A processing window for SEBM-fabricated pure tungsten is preliminarily determined by characterizing the surface morphologies of the melt pool, and the sample compression strength along the building direction is reported as 1.56 GPa [20]. Nevertheless, important questions, such as how to relate the mechanical properties of SEBM-ed pure tungsten samples to the microstructure and texture, remain to be fully elucidated. Moreover, no comparative analysis has been performed on the microstructural and mechanical characterizations of pure tungsten samples manufactured by SEBM or SLM.

In this work, we focus on some key issues of SEBM-ed pure tungsten samples, such as features of microdefects including cracks and pores, microstructure, texture, and mechanical properties. A thorough comparison on the microstructure and mechanical properties of pure tungsten samples manufactured by SEBM or SLM is performed, which is helpful in understanding and optimizing the additive manufacture of tungsten samples.

## 2. Materials and Experimental Procedures

### 2.1. Materials and SEBM Process

**Figure 1:**
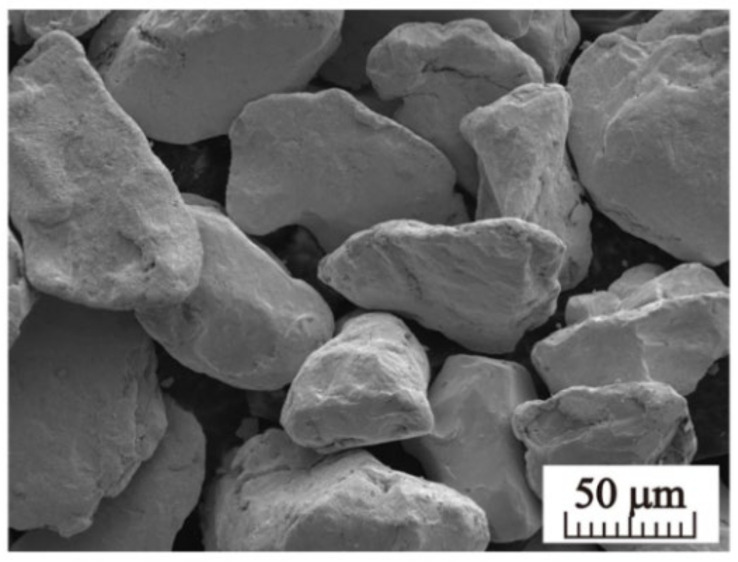
Morphology of tungsten powders used in the SEBM process.

Spherical powder particles of good flowability are most suitable for SEBM. However, owing to a large amount of powder required for SEBM and the corresponding expensive cost attributed to spheroidization of pure tungsten powder, polygonal pure tungsten powder of good flowability emerge as an alternative option. The Hall flow rate of polygonal pure tungsten powder particles with size of 65 μm–105 μm (Figure 1) is measured according to the ASTM B213 standard test method. Measurements with 50 g mass of powder require about 8.5 s, indicating fluidity good enough for SEBM.

**Figure 2:**
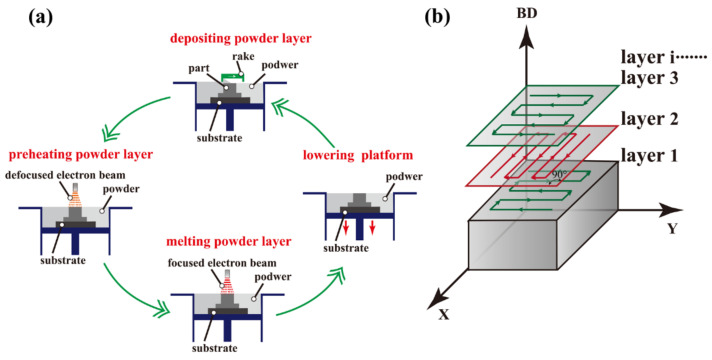
Schematic of the four-step process for building each layer in SEBM (**a**) and zigzag scanning strategy (**b**). BD, building direction.

The Arcam A2XX EBM system (Mölndal, Sweden) is used to fabricate the bulk pure tungsten samples with a minimum electron beam diameter of around 250 μm. The vacuum pressure in the chamber is kept below 0.2 Pa. The 316 stainless steel substrate plate is initially preheated to 1150 °C by fast scanning with a defocused electron beam to decrease thermal gradients during the building process. Then, a sample is fabricated layer by layer, and each layer is subject to a four-step process of depositing-preheating-melting a powder layer and lowering platform (Figure 2a): (1) depositing a powder layer onto the substrate plate by the rake, (2) preheating and slightly sintering the powder layer with a defocused electron beam, (3) selectively scanning and melting the preheated powder layer by a focused electron beam according to a schemed computer-aided system, and (4) lowering the building platform by a nominal layer thickness and repeating from step (1) for the next layer fabrication. Step (2) is essential to prevent so-called smoke events occurring in step (3), which lead to unfavorable powder spreading within the machine and eventual termination of the SEBM process [21]. A zigzag scanning pattern with an interlayer misorientation of 90° between layers is adopted (Figure 2b).

**Figure 3:**
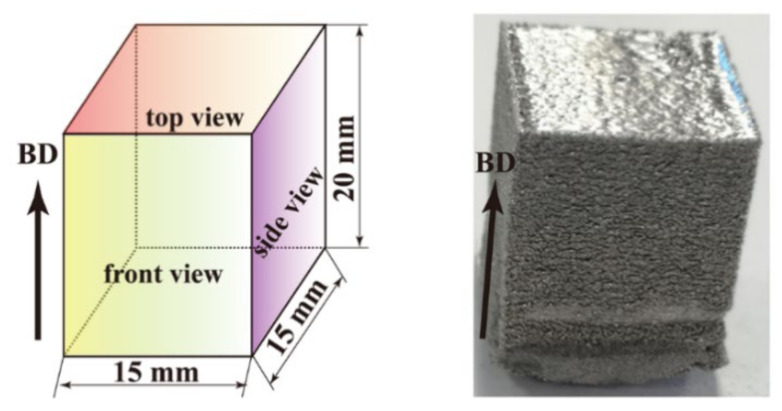
Pure tungsten cube (S4) of size 15 × 15 × 20 mm^3^ fabricated by SEBM.

Critical processing parameters impacting the input energy density in SEBM include the electron beam power *P*, scanning velocity *v*, powder layer thickness *t*, and hatch distance *h*. A combination of them gives the volumetric energy density *E* = *P*/(*h* × *v* × *t*), which is used to estimate the energy density input into the powder layer. The beam power is given by *P* = *I* × *U*, where *I* is the beam current and *U* is the voltage. The building processes exhibit features of lack of fusion, sufficient fusion, and over-melting as *E* increases. A lower *E* with a high scanning speed may induce the incomplete spread of the liquid metal and lead to the lack of fusion. An excessive *E* with a low scanning speed could result in the disturbance of the melt pool. Values of the processing parameters are listed in Table 1. The SEBM-ed pure tungsten samples have dimension of 15 × 15 × 20 mm^3^ (Figure 3).

### 2.2. Characterization

The SEBM-ed bulk pure tungsten samples are cut from the substrate using wire electrical discharge machining, then cleaned with acetone, alcohol, and water. For microstructure characterization, smaller specimens cut from the bulk components are mechanically ground, followed by electrolytic polishing with 2% NaOH solution at voltage of 20 V, then these specimens are examined with electron backscatter diffraction (EBSD) analysis. To detect the metallographic microstructure, the samples undergo electrolytic etching with 2% NaOH solution at voltage of 5 V and are examined using the Leica DM2700 M optical microscope (Wetzlar, Germany). Cylinder samples with length-to-diameter ratio *L*/*D* = 1.5 are cut from the fabricated components and the end faces are polished for compression tests at room temperature using an Instron 5585H universal testing equipment (Norwood, United States) at a strain rate of 10^−3^/s.

## 3. Results and Discussion

### 3.1. Metallurgical Defects

**Figure 4:**
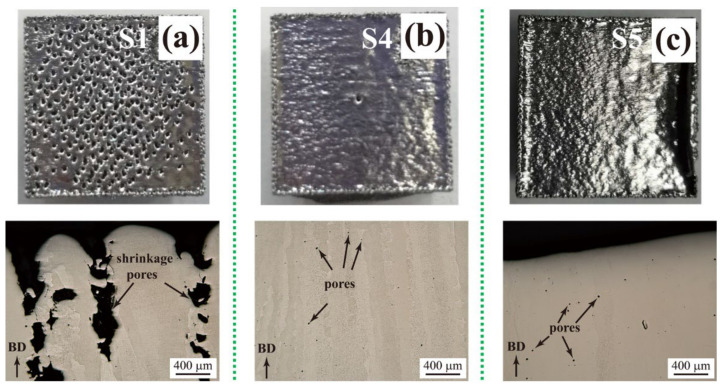
Three representative top surface morphologies (top row) and side view optical micrographs (bottom row) of the bulk pure tungsten samples (S1, S4, and S5) fabricated by SEBM with characteristics of lack of fusion (**a**), sufficient fusion (**b**), and over-melting or excess energy input (**c**). In (**c**), the surface periphery is higher than the central region. Lateral dimension is 15 × 15 mm^2^.

Three representative top surface morphologies and side view optical micrographs of the bulk pure tungsten samples fabricated by SEBM are shown in Figure 4. At high scanning velocity and low volumetric energy density, the liquid metal has no sufficient time to fully spread owing to the rapid solidification velocity, and evident shrinkage holes could be formed. As shown in Figure 4a, the corresponding fabricated samples show features of lack of fusion with large shrinkage holes having rough surfaces. As the scanning velocity *v* decreases to a proper value, samples of smooth surfaces and dense structures with few tiny pores are fabricated with sufficient fusion (Figure 4b). As *v* further decreases, the energy input becomes excessive, causing over-melting and unfavorable disturbance of the melt pool and leading to a higher surface periphery (Figure 4c). Although the energy inputs for the samples of sufficient fusion and over-melting are different, the pores there have similar size and number. The pore formation might be due to the trapping of gas such as residual oxygen within the powder particles, even the building process works in a high vacuum environment. A thorough and detailed mechanistic study on the formation of different surface morphologies is challenging and deserves further detailed investigations in the future. The effects of pores on the mechanical properties of the SEBM-ed samples are discussed comparing with SLM-ed samples in Section 3.3.

**Figure 5:**
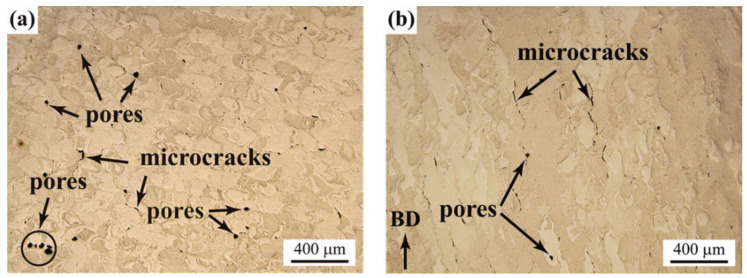
Optical micrographs of polished cross-sections (top (**a**) and front (**b**) views) for bulk pure tungsten samples fabricated by SLM.

No microcracks are seen; only pores (irregular shrinkage holes in the case of lack of fusion, and tiny spherical pores in cases of sufficient fusion and over-melting) exist in the SEBM-ed tungsten samples (Figure 4). In SLM-ed tungsten samples, microcracks are usually inevitable [6,8,9,10], as shown in Figure 5. A widely adopted aspect causing the microcrack formation in the SLM-ed samples is the high thermal stress generated by the high temperature gradient during the SLM process. That high thermal stress could cause tearing along grain boundaries, which are embrittled by the oxidation and resulting impurities [4,9,22,23]. It has been reported that a high concentration of oxygen and impurities segregate to the grain boundaries during the cooling process of melted tungsten in SLM [9,24], and the strength of pure tungsten and tungsten alloys are greatly affected by the oxides distributed at grain boundaries [22,25,26,27,28].

In SEBM, the vacuum environment prohibits the oxidation, and the high temperature gradient is lower compared to SLM. Therefore, no microcracks are formed in SEBM-ed samples. For example, in SEBM for tungsten fabrication the substrate is heated to 1150 °C by the defocused electron beam and that temperature is maintained throughout the building process. Each powder layer is also preheated. Therefore, the temperature gradient in SEBM is much lower than that in SLM for tungsten where the substrate can only be pre-heated up to 200 °C and there is no preheating for powder layers [8]. The lower temperature gradient in SEBM gives rise to a lower cooling rate, which in turn causes a much lower thermal stress [13,29,30] and low possibility of microcrack formation. Moreover, the ductile-to-brittle transition temperature (DBTT) of pure tungsten is in a range of 150 °C to 400 °C [1,31]. The sustained elevated temperature in SEBM could not only relieve the thermal stress [32], but also enable SEBM-ed tungsten samples above DBTT exhibiting plasticity to a certain extent. Overall, the crack formation in SEBM-ed tungsten is inhibited during the building process.

**Figure 6:**
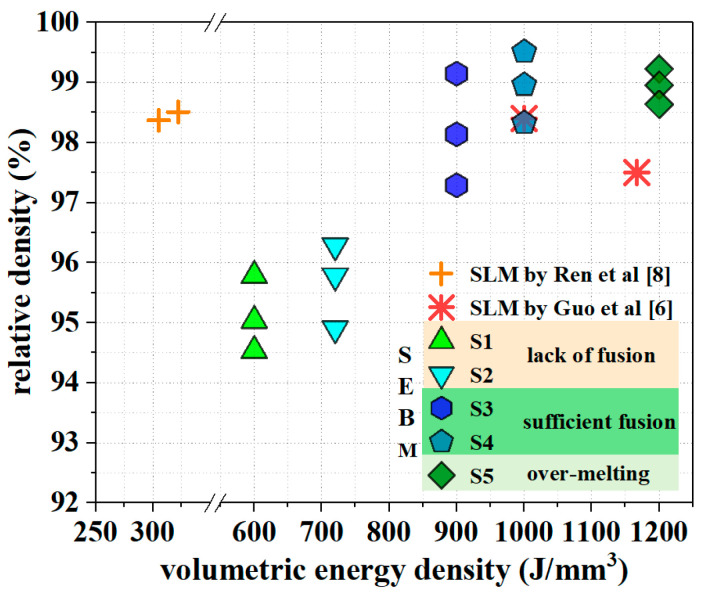
The relative density versus the volumetric energy density of the pure tungsten additive fabrication.

According to the Archimedes’ principle, relative densities of as-fabricated SEBM samples S1–S5 are measured. Having knowledge of the volumetric energy density *E* in Table 1, the relation between the relative density and the volumetric energy density is determined (Figure 6). It is shown that samples of either sufficient fusion or over-melting have high relative densities, and an S4 sample of sufficient fusion has the highest relative density of 98.93%. Though S5 samples have high relative densities, the associated irregular surface periphery, as shown in Figure 4c, limits the usage of S5 samples of over-melting as near-net shaped components.

In Figure 6, we also provide the values associated with SLM-ed tungsten for references. In our previous work on the additive manufacture of pure tungsten samples by SLM with spherical powder particles of 5 μm to 25 μm in diameter, the optimized SLM processing parameters include layer thickness of 30 μm, hatch distance of 0.08 mm, laser power of 300 W and scanning velocity of 300 mm/s at a laser spot size of around 70 μm [8]. As in the SLM-ed samples there only exist small microcracks and the crack number is small (Figure 5), the relative density of the SLM-ed samples is only slightly lower than the SEBM-ed samples of sufficient fusion with the absence of microcracks. In SLM and SEBM, the energy absorption depends on the powder particle size and shape as well as the physical features of laser and electron beams [33,34]. As all these parameters in the present work of SEBM and our previous work of SLM are significantly different, it is not surprising that the volumetric energy densities of the SLM-ed and SEBM-ed tungsten samples with similar relative densities could be significantly different (Figure 6).

### 3.2. Microstructure and Texture

**Figure 7:**
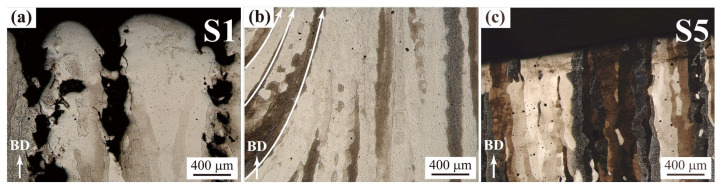
Columnar grain structures of as-fabricated pure tungsten samples of lack of fusion (**a**), sufficient fusion (**b**), and over-melting (**c**).

Columnar grains are observed in all SEBM-ed samples (Figure 7). It is known that the columnar grains grow along the maximum temperature gradient direction. In most cases, the building direction is parallel to the direction of the maximum temperature gradient, and one can see that columnar grains grow epitaxially along the building direction for SEBM-ed samples (as demonstrated in Figure 7a,c). In some cases, particularly at the sample edges surrounded by thick powder layers, the heat dissipation rate of the particles is much lower than that of the bulk, resulting in an evident deviation of the maximum temperature gradient direction from the building direction. That direction deviation is gradually reduced toward the bulk region, consistent with marked arrows in Figure 7b.

**Figure 8:**
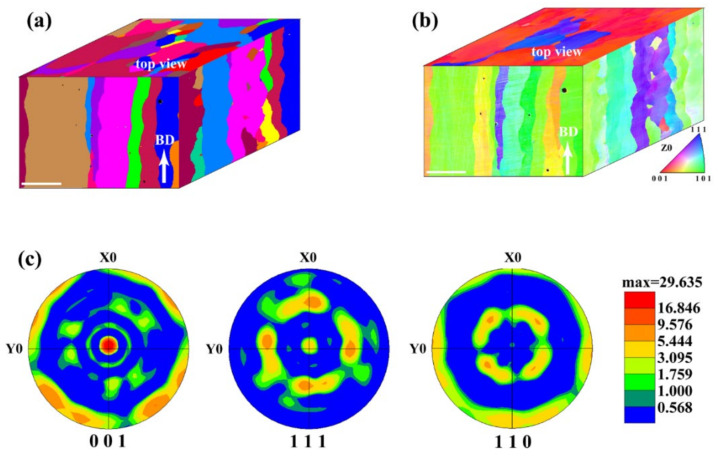
EBSD analysis of the SEBM-ed sample S4. (**a**) Grain size characterization, (**b**) inverse pole figure (IPF) coloring map, and (**c**) orientation pole figure taken from top view in (**b**). Scale bars in (**a**,**b**), 200 μm.

Microstructure and crystallographic texture analysis of the SEBM-ed sample S4 is performed using EBSD analysis. As shown in Figure 8, coarse grains appear equiaxed from the top view (Figure 8a), significantly different from the scattered checkboard pattern of SLM-ed samples reported in our previous work [8]. Long columnar grain structures along the building direction are observed in SEBM-ed samples (Figure 8a), indicating a stable melt pool during the SEBM process [20]. In contrast, columnar grain structures in the SLM-ed samples are relatively small (grain diameter about 125 μm in comparison with 320 μm for the SEBM-ed samples) and discrete [8], indicating disrupted epitaxy growth of columnar grains during SLM, which might be owing to the scanning strategy of a pattern with an interlayer misorientation of around 67° [8,10].

The IPF coloring map in Figure 8b and the pole figure in Figure 8c show that the SEBM-ed pure tungsten S4 sample has a strong <100> grain orientations. As pure tungsten has a body-centered cubic crystal structure, the columnar grains usually prefer <100> growth direction [35], consistent with Figure 8c. In the process of melting and solidification of tungsten, the epitaxial growth of columnar grains shows a strong rotated cube {100} <110> along the building direction. An unusual (111) texture has been found in SLM-ed tungsten, which is owing to the rotation of thermal gradient caused by the 67° rotation of scanning direction between layers [29,36]. The scanning direction with rotation of 90° between layers adopted in the present SEBM process may have little influence on the direction of maximum thermal gradient, and therefore the preferred <100> growth direction is observed in this work.

### 3.3. Mechanical Properties

**Figure 9:**
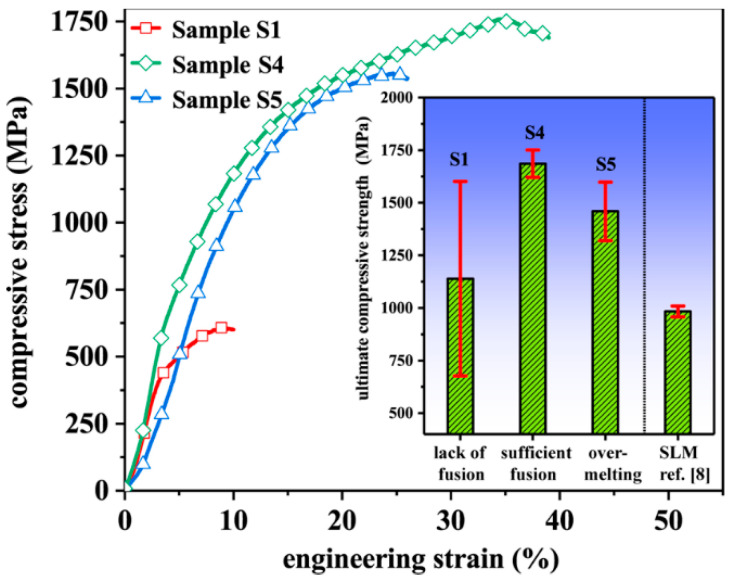
Compressive stress–strain curves for SEBM-ed pure tungsten samples S1, S4, and S5. Inset, ultimate compressive strengths of corresponding pure tungsten SEBM-ed bulk samples and previous SLM-ed sample.

Pore formation affects not only the density but also the mechanical properties of fabricated samples. Compression tests of cylindrical tungsten samples with features of lack of fusion (S1), sufficient fusion (S4), and over-melting (S5) are conducted at room temperature (Figure 9). The cylindrical samples have diameter of 3 mm and length of 4.5 mm. The average ultimate compressive strength for each sample is given in the inset of Figure 9.

Owing to the large and irregular pore structures in S1 samples (Figure 4a and Figure 7a), the corresponding ultimate compressive strength is as small as 0.61 GPa and the fracture engineering strain is as small as around 10%. Samples S4 and S5 of dense structures containing tiny pores (Figure 4b,c and Figure 7b,c) have a much larger ultimate compressive strength and fracture strain. Specifically, the ultimate compressive strength of the S4 sample (sufficient fusion) is 1.76 GPa with a fracture engineering strain of around 40%, both larger than reported values of 1.56 GPa and 18% in the literature [20]. Moreover, the volumetric energy density for the S4 sample is about 1000 J/mm^3^, significantly lower than that of 1440 J/mm^3^–3840 J/mm^3^ in ref. [20]. The compressive strength of optimized SLM-ed bulk pure tungsten samples is around 1.01 GPa [8], much lower than that achieved in the present work by SEBM, though the SLM-ed sample also has a high relative density of 98.51%. This observation suggests that the large ultimate compressive strength of SEBM-ed S4 sample mainly results from the absence of microcracks in fabricated samples, rather than the high relative density. In S4, samples the columnar grains are relatively coarse due to the long-time high temperature fabrication process, and grain refinement with addition of the carbide nanoparticles or oxide dispersion [9,22,23,26,37] could be employed to further improve the mechanical properties of fabricated samples.

## 4. Conclusions

Pure tungsten samples of high relative density and superior ultimate compressive strength are fabricated by SEBM at different volumetric energy densities. Samples with features of lack of fusion, sufficient fusion, and over-melting are identified. The corresponding metallurgical defects, microstructure, texture, and mechanical properties of the SEBM-fabricated samples are analyzed and compared with counterparts of the SLM-fabricated samples. The main conclusions are summarized as follows.

1. Pure tungsten bulk samples upon sufficient fusion using SEBM are fabricated with high relative density up to 98.93%. A superior ultimate compressive strength of 1.76 GPa higher than reported values in literature is achieved at a volumetric energy density of 1000 J/mm^3^.
2. Long columnar grain structures along the building direction are observed in the SEBM-ed samples, indicating a stable melt pool during the SEBM process; while relatively small and discrete columnar grain structures exist in the SLM-ed samples, indicating disrupted epitaxy growth of columnar grains during SLM.
3. Comparing with the SLM-ed pure tungsten samples containing micropores and inevitable microcracks, no microcracks, but only micropores, are found in the SEBM-ed tungsten samples of sufficient fusion. The absence of microcracks in the SEBM-ed tungsten benefits from the reduction in oxide precipitates due to the vacuum manufacturing environment and from the low thermal stress owing to the specific heating and preheating schemes in the SEBM process.
4. The SEBM-ed samples of sufficient fusion have the ultimate compressive strength (1.76 GPa) significantly larger than that (0.98 GPa) of the SLM-ed samples of similar high relative densities exceeding 98%.

## Figures and Tables

**Table 1:** Processing parameters for the fabrication of tungsten using SEBM.

	Beam Current *I*	Layer Thickness *t*	Hatch Distance *h*	Voltage *U*
	15 mA	50 μm	100 μm	60 kV
No. ^1^	Scanning Velocity *v* (mm/s)		Volumetric Energy Density *E* (J/mm^3^)	
S1	300		600	
S2	250		720	
S3	200		900	
S4	180		1000	
S5	150		1200	

^1^ S1 and S2, lack of fusion; S3 and S4, sufficient fusion; S5, over-melting.
